# The magnocellular system versus the dorsal stream

**DOI:** 10.3389/fnhum.2014.00786

**Published:** 2014-10-06

**Authors:** Bernt C. Skottun

**Affiliations:** Independent ScholarOslo, Norway

**Keywords:** contrast, magnocellular, ventral stream, dorsal stream, contrast-response

In his opinion paper, Breitmeyer (2014) sought to identify the contributions from the magno- and parvocellular systems to conscious and unconscious vision. One approach employed in this attempt was to study the effect of contrast. This was based on the well established finding that magno- and parvocellular cells differ in their contrast-response functions (Kaplan and Shapley, 1986): Magnocellular responses increase rapidly with contrast at low contrast levels but saturate at medium contrasts. Parvocellular responses, on the other hand, increase with contrast at a relatively constant rate. In his Figure 5, Breitmeyer makes the suggestion based on the effect of contrast, that visible priming reflects magnocellular activity whereas invisible priming reflects parvocellular responses.

In Figure 1 is shown (solid line) the function:

(1) $$r=(66.5\;c^{2.9})/(0.076^{2.9}+c^{2.9})$$

where *c* denotes contrast, which was assumed by Breitmeyer to reflect the contrast-response relationship of magnocellular neurons.

**Figure 1:**
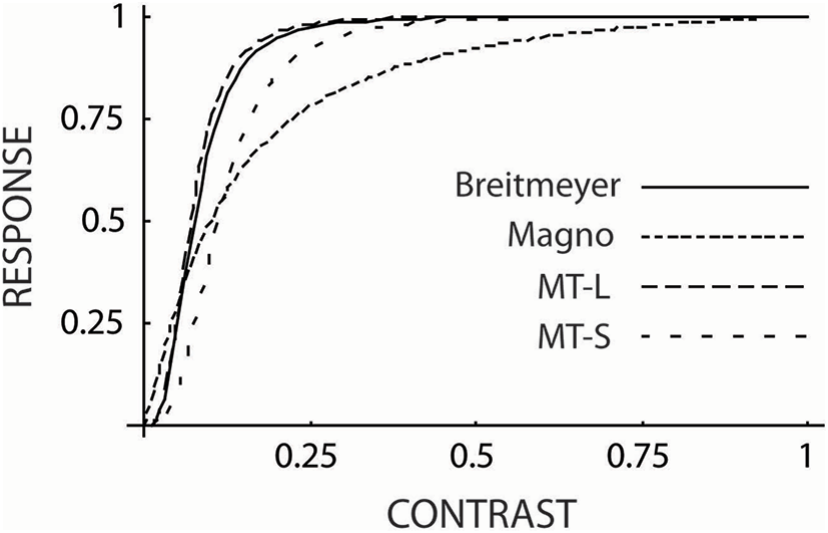
**The contrast-response curve from Breitmeyer (2014) (solid line) generated form Equation 1 which was by Breitmeyer attributed to magnocellular responses**. Also shown are contrast-response curves for magnocellular cells (dotted line) and for cells in cortical Area MT of the dorsal stream for large (MT-L) and small stimuli (MT-S). These functions were generated from Equation 2 (see text). As can be seen, the curve of Breitmeyer is much closer to the functions for the MT cells than to the one for magnocellular cells and is well within the range of MT cell responses to large and small stimuli.

Sclar et al. (1990) found that contrast-response data from visual neurons can be described by the equation:

(2) $$r=r_{max}\;c^{n}/(c^{n}+c_{50}^{n})$$

where *c* denotes contrast and the constants *r*_max_, *c*_50_, and *n* differ for the different types of neurons. For magnocellular cells these values were 52.7, 0.11, and 1.2, respectively (Sclar et al., 1990). By using these values in Equation 2 the dotted curve in Figure 1 was generated. The functions in the figure have been normalized so as to give a response of 1.0 for a contrast of 1.0. This is the only adjustment made. As can be seen, the function for the magnocellular cells from Sclar et al. does not provide a particularly close fit to the function of Breitmeyer.

This prompts the question: Would functions for other types of cells provide a better fit? Sclar et al. (1990) found that *c*_50_ (in Equation 2) for MT cells increases with decreasing stimulus size and that for very small stimuli it is comparable to that for magnocellular neurons. However, the exponent, *n*, was was found to be unchanged and the maximum response, *r*_max_, underwent only minor changes. The latter, however, is irrelevant in the present context since the responses are normalized. Using Equation 2 MT cell responses have been calculated for large and small stimuli. For large stimuli the constants *r*_max_, *c*_50_, and *n* were set to 36, 0.07, and 3.0 (from Sclar et al., 1990). In the case of small stimuli the value 0.11 (i.e., the value for magnocellular cells from Sclar et al.) was used for *c*_50_. As can be seen, the curves for MT cells (marked MT-L and MT-S for large and small stimuli, respectively) provide closer fits to the curve of Breitmeyer than does the function for magnocellular responses and the function of Breitmeyer falls between the two curves for MT cells.

Area MT is part of the dorsal cortical stream. The ventral and dorsal cortical streams represent two sets of cortical areas (Merigan and Maunsell, 1993). Many authors (e.g., Breitmeyer, 2014) have conflated the magno- and parvocellular systems with, respectively, the dorsal and ventral streams. This, however, faces some difficulties. In the case of the dorsal stream, although it clearly receives a substantial portion of its input from the magnocellular system it also receives sizable inputs from the koniocellular (Sincich et al., 2004) and parvocellular systems (Nassi et al., 2006). In the case of the ventral stream, as exemplified by Area V4, lesion studies have indicated that it receives about equally strong inputs from the magno- and parvocellular systems (Ferrera et al., 1994). Also, lesions placed in the dorsal and ventral streams have quite different effects from those placed in the magno- and parvocellular systems (Merigan and Maunsell, 1993). Figure 1 emphasizes that the magnocellular system and the dorsal stream (exemplified here by Area MT) also differ in regard to contrast-response functions.

## Conflict of interest statement

The author declares that the research was conducted in the absence of any commercial or financial relationships that could be construed as a potential conflict of interest.
